# The Rag Weed

**Published:** 1886-02

**Authors:** 


### HALL'S JOURNAL OF HEALTH

Vol. 33. FEBRUARY, 1886. No. 2.

### THE RAG WEED.

We notice in the looking over the proceedings of the State Medical Society of North Carolina, a very interesting paper on the virtues of "rag weed?" (*Ambrosia Artemesiœfolia*). To some, it is known as carrot and is known in this and all Northern States. The paper was read by Dr. J. H. Hill, of Goldsboro. He says it is as far as he knows, an indigenous annual weed, always following a wheat crop, flowering about the middle of August. He says of it: "Not professing to be a botanist, I cannot give a clearer description of it." Dr. Francis Peyre Porches in "Romances of Southern Fields and Forests," says the rag weed grows in cultivated lands, and pastures; collected in St. John's Charleston district. It is common in most of our wheat fields and is thought to be too plentiful for the farmer generally. The plant is used in fevers in Maryland as a substitute for quinine; a tincture is made of the juice given with whiskey. It is very bitter, and is thought to be useful." It is used as a common domestic remedy for nose-bleed, epistaxis, in Rowan County, where I first heard of its properties and witnessed its effects, as long ago as 1857, where I frequently saw it used to arrest hæmoptysis in the case of a negro carriage-driver of my father-in-law. This negro was a frequent sufferer from the complaint, and always carried a portion of the weed in his pocket, and being attacked would take a chew of it, swallowing the juice, with almost immediate relief and was told that he had thus used it for many years. I also saw it was used as a stystic and applied locally in case of violent epistaxis, as in a boy ef ten or twelve with immediate effect, when I thought, from the volume of the hemorrhages, the posterior naus would have to be pluged. As I stated before, it was the common domestic remedy through that country for these complaints, as I was informed by Drs. Whittaker and Summerell, the latter of whom confirmed my statement at the Tarborough meeting.

In my previous paper I referred to several cases in which I had used the remedy, but more particularly to the following. On the

23d of September, 1882, I was called to see a child, son of Mr. William Smith, aged about six years. Found him bleeding from the nose, gums and bladder, passing a short time after I arrived nearly a pint of what I took to be pure blood from the bladder; skin tallowy, and legs and body covered with spots of puspure, from the size of pin-point to that of a silver dollar. He had been bleeding for several days, and had been seen by two other physicians, who had given him ergot, lead and opium, aromatic sulphuric acid, etc., etc., with no good effect. I was also informed that two sisters of the father had died from hemorrhage, and that the family all had difficulty in arresting what to others would seem timid bleedings. I thought it a very bad case, and so informed the father, but told him that if I could find a certain weed I knew of, I thought possibly I might arrest it. I soon found a branch of it in a fence corner, and gathered a handful of the leaves, which, I put into a cup of boiling water and made a strong tea, of which I gave a large tablespoonful to the boy, at first every half hour, and then at longer intervals. I plugged the nostrils with cotton saturaed, the per. sulph. ferri. Within fifteen minutes the effects of the medicine were seen. The oozing from the gums ceased, there was none from the nose, and as I was afterwards informed, when he made water it was only slightly colored with blood. I remained with the case four or five hours, and when I left prescribed mur. tr. ferri in a three drop dose in a glass of water three or four times a day. The next day the father came to town, called at my office perfectly delighted, paid his bill, and since then my reputation has been made in that neighborhood, although I did cure the case with "yarbs." Since then the boy has been perfectly well, except that occasionally in the spring there is a tendency to bleeding from the nose—always arrested though, at once by the ragweed, of which the father is always sure to have a good supply on hand."

"On the 5th of March, 1855, I was called hurriedly to see Mrs. W., of Goldsborough, before breakfast. Her husband came for me very much excited, stating that his wife was bleeding to death, the blood just streaming from her mouth; that she was the patient of another physician, but that he was absent in Washington at the inauguration. I put in my pocket a package of rag weed, of which I always have a supply, gathered when it is in bloom, and went with him at once. I found the lady propped up in bed with a chamber-mug by her side nearly full of blood, which she was spitting up by the mouthful—evidently a case of hæmoptysis. I pulled off a portion of the weed and requested her to chew it and swallow the juice while I was having a decoction prepared, which, as soon as it was cold,

I gave her in wineglassful doses, assuring her it would do the work. In an incredibly short time the hemorrhage ceased. She was directed to continue the medicine at interval of three or four hours. I called in the evening and found her comfortable—no blood except when she would cough up a few hard coagula. Called the next morning, found her up eating a hearty breakfast of beefsteak and buckwheat cakes; no sign of blood. A day or two afterwards she sent to my wife and asked her to please send her a little of that "weed," she would not be without it for anything. I put her on tincture of iron and she has had no trouble since."

Other cases are given but they are of the same nature and seem to prove beyond dispute the virtue of this simple weed in very serious cases. The Dr. then concludes his remark with the following very sensible suggestion: "Now, Mr. President, having detailed the virtues of one of our indigenous remedies, I would like to say a few words on a subject in which I take a deep interest, although I may lay myself open to the sarcasm of being an 'old woman doctor' a 'yarb doctor,' etc. But how many of the common domestic remedies of our country are valuable! I myself could name a good many, and I doubt not but that every gentleman has some remedy in daily use which is not to be found in the materia medica —some remedy which he knows from experience to be valuable although he may have have gotten it from an old woman maybe an old Negro. How many of us during the war were forced to use the common remedies and with what joy we hailed the advent of Dr. Francis Peyer Porcher's "Resources of Southern Fields and Forests." Suppose each member of the North Carolina Medical Society should contribute a short paper giving his experience with some home remedy, trifling, perhaps, in itself, to him, but collectively of what incalulable value to the profession, particularly so to country practitioners."

